# Translation regulation and pain special issue editorial for neurobiology of pain

**DOI:** 10.1016/j.ynpai.2018.05.001

**Published:** 2018-05-30

**Authors:** Theodore J. Price

**Affiliations:** The University of Texas at Dallas, School of Behavioral and Brain Sciences, 800 W Campbell Rd, Richardson, TX 75080, United States

The field of translation regulation and neuronal plasticity started with a remarkably simple observation, made by Steward and Levy (1982): dendritic spines are relatively devoid of ribosomes, except at the base of dendritic spines. Based on their analysis of electron micrographs from rat brain, these pioneering neuroscientists hypothesized that local translation at the base of dendritic spines likely contributes to synaptic plasticity. They could not have been more correct. Thousands of papers have now been published on this topic and we now understand the nuances of how localized translation controls synaptic plasticity in remarkable molecular detail (Costa-Mattioli et al., 2009).

What is less well known is that the field of localized translation in axons preceded the wide-spread recognition that dendritic translation control is involved in synaptic plasticity (Koenig, 1967, Koenig, 1979, Alvarez et al., 2000). One of the obstacles to progress in this area was the difficultly in reliably identifying ribosomes in axons of mammalian neurons. New imaging techniques and next generation sequencing have now led to important advances in this field. The last decade has seen an explosion of work on mechanisms of localized translation control in the axonal compartment and its contribution to axon development, regeneration, and repair (Costa and Willis, 2017, Khoutorsky and Price, 2018). This work has been paralleled by the growing recognition that axonal translation plays a crucial role in the sensitization of nociceptors, which is a hallmark of the development of chronic pain. It is particularly striking that this mechanism of sensitization is likely conserved from *Aplysia* (Weragoda et al., 2004, Weragoda and Walters, 2007) to mice (Melemedjian et al., 2010) to humans (Obreja et al., 2017).

This special issue of *Neurobiology of Pain* contains several review articles that give a comprehensive viewpoint on what we currently know about translation regulation and pain, how this knowledge can be used to develop completely new kinds of pain therapeutics and where the field might be headed. This special issue also contains two primary research articles that give new insight into how translation regulation is involved in pain sensitization in the peripheral and central nervous systems. I am grateful to all of the authors who committed to share their deep knowledge of the subject through this special issue on Translation Regulation and Pain.

This is the first special issue of *Neurobiology of Pain*. It is fitting that translation regulation is the topic of the first special issue of this journal because it can be persuasively argued that this subfield of pain research began in the laboratory of the Editor in Chief and Founder of *Neurobiology of Pain,* Fernando Cervero*.* When I was a postdoctoral fellow in Dr. Cervero’s lab working on those first studies in this area of research (Price et al., 2006, Price et al., 2007), I doubt that either of us anticipated that this would turn into such a robust area of enquiry for the field. I certainly did not! Ten plus years on from those original publications it is extraordinarily gratifying to have the opportunity to contribute such a rich volume covering such a broad range of new scientific knowledge to the field. It is even more rewarding to do so under the editorial guidance of my mentor and friend, Fernando.

## Conflict of interest

The authors declare that they have no conflicts of interest.
